# Correlation between vitamin D blood levels and implant success: a prospective cohort study

**DOI:** 10.1186/s12903-026-09885-0

**Published:** 2026-10-14

**Authors:** Shaimaa Saieed Nasr, Ramy Moustafa Moustafa Ali, Rania Ihab Younis, Shahinaz Gamaleldin Mohamed Elashiry, Nabil Ahmad Ibrahim, Mohamed Hamdy Mahmoud Ismail

**Affiliations:** 1 Department of Oral Medicine and Periodontology, Faculty of Dentistry, Fayoum University, Fayoum, Egypt; 2 Department of Oral Medicine and Periodontology, Faculty of Dentistry, October University for Modern Science and Arts (MSA), Giza, Egypt; 3 Department of Prosthodontics, Faculty of Dentistry, Fayoum University, Fayoum, Egypt; 4 Department of Prosthodontics, College of Dentistry, king Faisal University, Al Ahsa, Kingdom of Saudi Arabia; 5 Faculty of Dentistry, MSA University, Giza, Egypt; 6 Department of Periodontology Faculty of Dentistry, German University in Cairo (GUC), Cairo, Egypt; 7 MSA University, Giza, Egypt

**Keywords:** Vitamin D, Early implant failure, Osseointegration, Implant failure

## Abstract

**Background:**

Vitamin D is a fat-soluble hormone that can be produced in the skin by Ultra-Violet (UV) light exposure or acquired through diet. Vitamin D is currently of relevance to dentists placing dental implants because it plays a role in bone metabolism and controls the immune system. There is some evidence that the osseointegration process of dental implants is favorably correlated with the proper concentration of this prohormone. Several studies have demonstrated that vitamin D may be crucial for both the integration of the implant into bone tissue and the postoperative tissue repair process.

**Aim:**

The aim of this study was to find out if there was a correlation between serum vitamin D levels and implant success.

**Methods:**

This study was conducted on 100 patients divided into two equal groups who received single dental implant to restore single missing tooth. After implant placement, patients were not advised to receive any supplements for 3 months. Patient’s serum level of vitamin D was tested after 3 months of implant placement (osseointegration period). Clinical and radiographic assessment of dental implants were performed after 3 months postoperatively.

**Results:**

Statistical analysis showed no statistical significance results (*P* value 0.277) regarding the positive correlation between vitamin D levels and implant success/ failure, it also showed no statistical significance (*P* value 0.663) regarding the relationship between Vitamin D levels and implant success/failure based on the implant location.

**Conclusion:**

Serum vitamin D level has no direct effect on the success or failure of dental implants.

## Introduction

Dental implants are considered a reliable approach for restoring missing teeth for functional and esthetic purposes. Success of dental implants depends mainly on osseointegration, which is the direct contact between bone and implant to achieve implant stability [1].

Ossseointegration depends on several factors, such as implant design, implant surface treatment, surgical protocol or prosthetic design, and the qualitative and quantitative properties of the patient’s bone [1].

Many authors described the criteria of implant success and failure. Albrektsson et al. described that criteria to describe implant success or failure depend on many observations as presence of radiolucent margins around the implant, pus or fluctuation around the implant, pain, implant micro or macro-mobility, and presence of bone loss around the implant > 1.5 mm during healing phase [2].

Resonance frequency analysis is a well-known non-invasive technique for the measurement of implant stability. Several authors used this technology to detect implant success\failure. Andersson et al. conducted a retrospective study to correlate between implant stability and implant success based on Implant Stability Quotient (ISQ). They concluded that any implant with lower than 60 ISQ value threshold at the time of prosthetic loading is considered a failed implant and has a very poor survival rate [3].

Noteworthy, early implant failure could occur due to failure of osseointegration or peri-implantitis. These events happen during the early stages of osseointegration (with the first 2–3 months of dental implant placement). Therefore, these are pre-functioning implant failures [3].

Early implant failure may occur despite strict adherence to surgical protocols and the use of optimal implant materials, suggesting that patient-specific risk factors may contribute to impaired osseointegration [4].

There are several factors that could improve results of osseointegration, such as bone augmentation, osteogenic biological coating, and pharmacological agents. Cholecalciferol supplements (Vitamin D3) was reported to show benifical results on bone density [5].

Vitamin D is a fat‑soluble biological molecule, which is obtained through Vitamin D‑rich diet or generated in the skin through exposure to ultraviolet light. Vitamin D‑binding protein is responsible for transporting Vitamin D from the skin or intestine to the liver, where it is metabolized and yields 1,25‑dihydroxyvitamin D, or 1,25(OH)2D. Next, 1,25 (OH)2D will produce the active form of Vitamin D in the kidneys: 1,25‑dihydroxyvitamin D, also known as 1,25(OH)2D or calcitriol, which is responsible for binding to Vitamin D receptor (VDR) [6]. The VDR is a ligand‑dependent transcription factor of the nuclear receptor superfamily, which is involved in xenobiotic metabolism [6].

Vitamin D deficiency has become very common and can result from inadequate dietary intake together with insufficient exposure to sunlight. Therefore, vitamin D has been shown to play a vital role in bone mineral homeostasis by stimulating intestinal absorption of calcium and phosphate [6]. Serum level of Vitamin D in the 25(OH) D form is the most precise method of determining Vitamin D status: A patient with < 10 ng/mL is considered to be Vitamin D deficient; one with 10–30 ng/mL is considered to have low levels of Vitamin D. The optimal blood level of Vitamin D is a value > 30 ng/mL [7, 8].

## Materials and methods

### Study settings and ethical considerations

This prospective study was conducted on 100 patients collected from the implant clinic, faculty of Dentistry, MSA University who need to restore missing teeth with dental implant after reviewing and approval of the ethical committee of faculty Dentistry MSA University following the Declaration of Helsinki guidelines with approval number (REC-D 33512-5).

### Inclusion criteria

1. Patients with missing single tooth and requires single implant placement
2. Patients free from any systemic disease
3. Patients who approve to be included in the trial and sign the informed consent
4. Patients without bony defects
5. Age range of the patients was 18 to 45 years old

### Exclusion criteria

1. Heavy smoker patients (> 10 cigarettes\day)
2. Patients receiving chemotherapy or radiotherapy

### Preoperative assessment

- Clinical assessment of inter-arch space and soft tissue quality
- Radiographic assessment: via Cone Beam Computed Tomography (CBCT) for the implant site to measure the bone width, height, and to detect the relation to the neighboring vital structures

### Surgical procedure

- Crestal and sulcular incisions were performed at the implant site.
- Osteotomy was prepared under copious irrigation following manufacturer’s instructions.
- An implant of suitable size was inserted “Neobiotech IS II, South Korea”.
- Closure of surgical wound was achieved.
- Patient’s vitamin D level was tested after 3 months of osseointegration period regardless of implant’s success or failure.

### Post-operative instructions

- After implant placement, patients were instructed not to receive any supplements for 3 months.

### Postoperative assessment after 3 months

- Clinical assessment was done by measuring the secondary implant stability by Osstell “Osstell^®^, integration Diagnostic AB, Goteborg, Sweden “expressed ISQ values. Also clinical inspection was done to assess the presence of any pain, infection, or discomfort.
- Radiographic assessment was done via periapical digital x-ray to detect any signs of radiolucency or bone loss around the implant.

### Criteria of implant failure at the time of prosthetic phase: table 1

**Table 1:** Criteria of implant failure

Clinical	Radiographic
Pain	Crestal bone loss >2 mm
Swelling	Radiolucent margin around the implant
Macro-mobility	
Low ISQ values < 30	

### Sample size calculation

The sample size was calculated based on the primary outcome and Mangano F et al. [9] using the formula n=(Zα + Zβ)2σ2/δ2. Assuming a significance level of 0.05, a statistical power of 80%, an expected standard deviation of 8.0, and an effect size of 0.43, the minimum required sample size was 44 patients per group. With 10% dropout rate, the sample size was increased to 50 participants per group.

## Results

### Statistical analysis

Statistical analyses were conducted using SPSS, version 26. The dataset included 100 individuals who underwent single tooth restoration via dental implant, categorized into two groups based on the outcome: successful and failed implants.

### Descriptive results

The analysis was done on 100 individual (46 male and 54 females) with age ranged from 28.5–35.1(5.6) who had single implant in the time from April 2023 to September 2024. The implant’s distribution for the failed group was (27 implants at lower posterior area, 14 at upper posterior area, 9 implants at upper anterior area) while for the successful group it was (23 implants at lower posterior area, 17 implants at upper posterior area, and 10 implants at upper anterior area). Tables 2 and 3.

**Table 2:** The following table provides the subgrouping of failed and successful implants regarding the level of vitamin D deficiency / sufficiency

Parameter	Failed Implants (*n* = 50)	Successful Implants (*n* = 50)
Mean ± SD	24.07 ± 10.88	26.21 ± 7.93
Median (IQR)	25.53 (15.81–30.22)	26.51 (20.21–30.62)
Min–Max	5.40–50.40	7.50–45.11
95% CI	21.02–27.11	24.02–28.41

**Table 3:** The following table provides sensitivity and specificity of vitamin D level and implant success\ failure

Threshold	What It Predicts	Sensitivity	Specificity	Problem
18.5 ng/mL	SUCCESS	88.0%	36.7%	Misses 63.3% of failures
17.1 ng/mL	FAILURE (inverse)	92.0%	32.7%	Misses 67.3% of failures

#### The association between vitamin D levels and implant success/failure

**Fig. 1:**
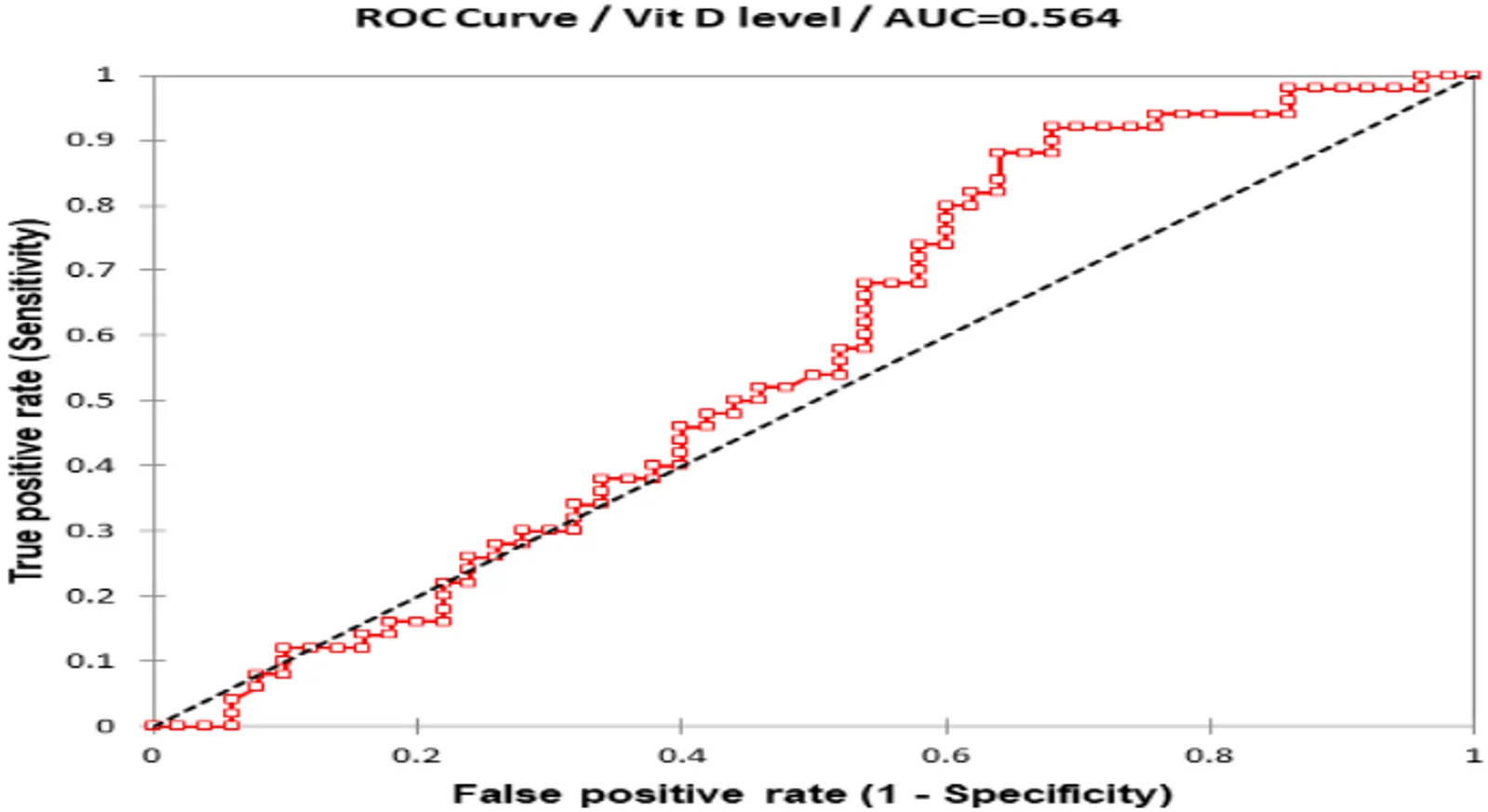
ROC analysis

The ROC curve was used to confirm the positive correlation between vitamin D levels and implant success/ failure. The ROC analysis (AUC = 0.564, *P* = 0.277) demonstrated no statistically significant predictive Fig. 1; Table 4.

**Table 4:** The following table provides reported vitamin D levels in both groups

Vitamin D Level	Failed Implants (*n* = 50)	Successful Implants (*n* = 50)	Total (*n* = 100)
≤ 17.1 ng/mL	17 (34.7%)	5 (10.0%)	22 (22.2%)
17.1–18.5 ng/mL	3 (4.1%)	3 (6.0%)	6 (5.1%)
≥ 18.5 ng/mL	30 (61.2%)	42 (84.0%)	72 (72.7%)

#### Relationship between vitamin D levels and implant success/failure based on the implant location

The Chi-square test was used and yielded no statistical significance (*P* value 0.663) regarding the relation between implant success / failure to the vitamin D level.

## Discussion

Dental implants have established themselves as a highly reliable modality for tooth replacement, characterized by high long-term survival rates [10, 11]. The ultimate goal of implant placement is predicated on the success of osseointegration during the initial healing phase [12, 13]. This biological process is influenced by a complex interplay of several factors, including surgical technique, clinician experience, the timing and nature of prosthetic loading, the chemical and physical properties of the implant surface, and patient-specific factors such as bone quality, local inflammation, tobacco use, and systemic nutritional status [11, 14–18].

Among patients’ systemic factors is vitamin D, which is a steroid hormone synthesized via UV exposure or acquired through dietary intake. It undergoes metabolic conversion in the liver by the hydroxylated enzyme CYP27A1 to form 25-hydroxyvitamin D3 [25(OH)D]; accordingly, it plays a critical role in skeletal homeostasis and bone metabolism [19]. 

Clinically, vitamin D status is quantified by serum concentrations of 25(OH)D, where levels below 10 ng/mL represent severe deficiency, 10–24 ng/mL indicate moderate deficiency, and 25–80 ng/mL are considered within the normal range [20]. Given that bone metabolism directly governs protein rearrangement and mineral deposition at the implant interface, low vitamin D serum levels may impede the formation of new bone and increase susceptibility to peripheral infections, potentially compromising the primary stability of the fixture [21].

Our current study found no statistical significance regarding the direct relation between serum vitamin D levels and early implant failure. This was in accordance to a systematic review that was conducted by Alsulaimani et al., and it was difficult to find a direct association between low serum vitamin D level and early implant failure [22].

On the other side, a systematic review included human studies on direct relation between serum vitamin D level and osseointegration. Some studies have found that low vitamin D levels have negative impact on healing after implant placement and its high levels might have positive impact on osseointegration, reducing bone loss, dental implant survival, and consequently on the early implant failure therefore, early implant failure rate was found to be higher in patients with vitamin D deficiency. However, other studies failed to report this direct association [23, 24].

Additionally, Mangano et al. discovered a four-fold increase in early implant loss in patients with low serum Vitamin D levels [25]. Similarly, another study by Pereira et al. have exposed that significant association between low Vitamin D level and poor osseointegration, could be related to potential genetic predisposition that could affect the results [26]. However, majority of studies in a systematic review Buzatu et al. .reveals no significant association between Vitamin D levels and the osseointegration of dental implants, and this could be the high heterogeneity among the included studies in the literature [27].

Moreover, a study included 90 patients and divided into groups based on their serum vitamin D levels. It was found that early implant failure was obvious in 2 out of 15 patients in the deficient group, and 3 out of 44 patients in the insufficient group, while 1 out of 31 in the adequate group. Therefore, there could be progressive reduction in early implant failure rates with increasing vitamin D levels [28]. This was aligned with a review by Miron et al. that have shown moderate but clinical correlation between vitamin d deficiency and implant failure [29].

Toy VE et al. conducted a clinical study on 42 patients to find out the correlation between low vitamin D levels and increased level of RANK-L levels. They found a highly statistical significance of increased level of RANK-L and implant failure which concludes that low vitamin D levels may significantly affect implant success which does not coincide with our current results. Despite our results showing moderate effect between vitamin D level and implant success yet we could not reach a statistical significance [30]. 

It was difficult to correlate the results of the current study with other articles in the literature because none of the existing systematic reviews demonstrated homogeneity of results concerning the influence of vitamin D on early implant failure. There were no consistent findings in the literature. Moreover, few articles were performed on human subjects, and the majority were animal studies with different regenerative capacity. Therefore, further studies on the association between serum vitamin D level could enhance to find clear conclusion regarding the correlation between them.

## Conclusion

Following the limitations of this study we concluded that, vitamin D level has no effect on dental implant success\ failure.

## Data Availability

Raw data are available upon request.

## Abbreviations

UV: Ultra Violet
ISQ: Implant Stability Quotient
1,25(OH)2D: 1,25 dihydroxyvitamin D
VDR: Vitamin D Receptor
CBCT: Cone Beam Computed Tomography
SPSS: Statistical Package for Social Sciences
SD: Standard Deviation
CI: Confidence Interval
ROC: Receiver Operating Characteristics
AUC: Area Under Curve
RANK-L: Receptor Activator of Nuclear factor kB Ligand
